# Advancing continuous enzymatic hydrolysis for improved biomass saccharification

**DOI:** 10.1186/s13068-025-02680-z

**Published:** 2025-07-25

**Authors:** Roman Brunecky, Yudong Li, Stephen R. Decker, Michael E. Himmel

**Affiliations:** 1 Biosciences Center, National Renewable Energy Laboratory, 15013 Denver West Parkway, Golden, CO 80401 USA; 2 Catalytic Carbon Transformation and Scale-Up Center, National Renewable Energy Laboratory, 15013 Denver West Parkway, Golden, CO 80401 USA

**Keywords:** Biomass, Cellulase, Enzyme hydrolysis, Saccharification, Biomass sugars, Lignocellulose, Sugar depot

## Abstract

**Background:**

A deployable, continuous enzymatic hydrolysis (CEH) process can address cost and commercialization risks associated with second-generation (Gen2) biorefinery sugar/lignin/ethanol production while contributing to energy supply and security. Developments in commercial enzymatic hydrolysis formulations targeting Gen2 pretreated biomass such as deacetylated mechanically refined (DMR) biomass necessitate a reassessment of the existing hybrid simultaneous saccharification and fermentation (SSF) approach. Notably, the practice of "finishing hydrolysis" in SSF has become problematic with the introduction of oxidative enzymes, such as lytic polysaccharide monooxygenases (LPMOs), into commercial cellulase formulations as these require specific redox conditions and cofactor. Moreover, continuous SSF has not been demonstrated at commercial scale, limiting deployment and the associated economic benefits to farmers, producers, and support industries.

**Results:**

Continuous enzymatic hydrolysis (CEH) was demonstrated at bench scale to enable optimal saccharification performance of deacetylated mechanically refined (DMR) pretreated biomass. Diafiltration was demonstrated to retain pretreated biomass solids and enzymes for continuous reaction while removing solubilized product sugars in situ. A significant breakthrough afforded by the CEH process is its ability to achieve equivalent endpoint conversions with approximately 50% lower enzyme loading. Yields of glucose and xylose were increased ~ 15% and ~ 4%, respectively, over batch hydrolysis. Unlike SSF using yeast or *Zymomonas*, CEH allows precise optimization of pH, temperature, oxygen tension, LPMO mediator concentration, and removal of end-product inhibitors.

**Conclusions:**

Advanced CEH holds promise as a transformational, process-intensified, and cost-effective method for producing soluble clarified biomass sugars and insoluble lignin-rich streams. Enhancing saccharification performance, optimizing operating parameters, and employing membrane filtration will help overcome existing challenges and enable the efficient production of valuable biomaterials from lignocellulosic biomass.

## Background

The common conversion routes being pursued today at commercial scale for production of lignocellulosic sugars/lignin-based Sustainable Aviation Fuels (SAF) and chemicals are based on the basic biological strategy (i.e., pretreatment followed by enzymatic hydrolysis and fermentation) or the chemical strategy (preprocessing followed by chemical catalysis using solid catalysts). For an envisioned biochemical lignocellulose feedstock-based biorefinery, enzymatic hydrolysis of pretreated biomass is the rate limiting deconstruction step [1].

The development of commercially successful second-generation (Gen2) biorefineries is crucial for enabling a new bioeconomy. These refineries focus on converting lignocellulosic biomass, comprised of cellulose, hemicellulose, and lignin, into valuable products, such as sugars, lignin, and ethanol [2]. However, a major challenge in this process remains the enzymatic hydrolysis of pretreated biomass feedstocks to release fermentable sugars [3, 4]. Unlike the more readily hydrolyzed starch, native cellulose and hemicellulose polysaccharides in Gen2 feedstocks are highly resistant to enzymatic deconstruction and require combinations of thermal, chemical, and mechanical treatment prior to enzyme addition [5, 6]. Traditional batch pretreatment processes are tradeoffs between extent of hydrolysis and sugar loss through degradation reactions. Dilute acid-based pretreatment processes, for example, are known for high capital cost (i.e., custom reactors made from expensive materials of construction); as well as high operating temperatures, pressures, and required enzyme loadings. The sugar production rates and yields of these pretreatments are also low [7–10]. The recently patented deacetylated mechanically refined (DMR) pretreatment process (US Patent No. 12,129,342 B2), in contrast, is conducted at low temperature and pressure, alkaline pH, produces no degradation products, and has no requirement for costly custom reactors made from acid-resistant alloys [11].

Classic Simultaneous Saccharification and Fermentation (SSF) of pretreated lignocellulosic biomass remains problematic in continuous mode. The SSF process was originally developed and demonstrated in batch mode; however, all commercial biorefinery models are based on fully continuous operation and thus continuous fermentation of soluble sugars is well-known. Even with an effective pre-saccharification step, deployable as a continuous paddle type pug mill mixer designed to transport slurries during the mixing/reaction process, fermenting slurries in continuous mode remains problematic. Optimal operating parameters such as pH, temperature, and oxygen tension differ between fermentative microbes and lignocellulose degrading enzyme formulations [12]. Sugar utilization and production rate mismatches can lead to inefficiency in the process and non-specific unproductive binding of enzymes to biomass solids reduces hydrolysis rate and efficiency and increases the enzyme loading required. An advanced solution is to fully hydrolyze the lignocellulosic polysaccharides to soluble sugars in a saccharification step prior to their addition to classical fermentation and abandon the SSF (or hybrid SSF) modality. Membrane filtration recovery of soluble sugars produced by hydrolytic enzymes has the potential to retain the kinetic advantages of SSF but avoid the difficulties of maintaining a continuous SSF process.

Enzymatic production of sugars, primarily glucose and xylose, from biomass relies on a combination of biomass pretreatment to make the polysaccharides accessible, the activity and amount of enzyme applied, the biomass concentration, and the time of hydrolysis. Pretreatment processes vary widely but are typically short in duration; half-day or less (hydrothermal/steam explosion and DMR). Enzymatic hydrolysis (EH), in contrast, may require as many as 7 days, though recent advances in cellulase activity engineering could reduce that [13–17]. Note that enzyme loading rates are best measured on a mass basis, i.e., gram protein per gram biomass or glucan, as true activity units are almost impossible to measure due the complexities of the activities involved and the wide variation of substrates [18, 19]. While convenient, the classic Filter Paper Unit measure of cellulase activity has little relevance to digestion of complex biomass as the measurement is based on limited hydrolysis (3.6%) of a non-relevant substrate (Whatman #1 filter paper) [20, 21]. Higher enzyme loadings are faster, but more expensive. At high loadings, inhibitory product levels build up faster and enzyme efficiency decreases as substrate is hydrolyzed, effectively decreasing the available substrate level. The remaining substrate is also increasingly more recalcitrant, as the enzymes act on the easily digestible and accessible fractions first. Biomass loading impacts the rate and extent of EH, as high biomass loadings result in higher sugar concentrations which will inhibit the hydrolases. In addition to introducing problems with mixing and pumping, high biomass loading will slow down and then stall the digestion as these end products rise to inhibitory levels. Because of this decrease in activity as the hydrolysis proceeds, extending the time of digestion has a decreasing rate of return, with longer times generally resulting in lower rates and decreasing efficiency.

To address these challenges, a continuous enzymatic digestion approach for DMR treated corn stover slurries was developed, which diverges considerably from the traditional batch pre-saccharification and SSF approach [22]. Using DMR pretreated biomass [23], this method has demonstrated significant advancements in saccharification performance. The CEH process allows for the precise control of enzymatic parameters, such as pH, temperature, and oxygen tension, which are vital for optimal enzyme activity and stability with state-of-the-art commercial cellulase formulations. By removing solubilized sugars and low molecular weight lignins using diafiltration and molecular weight cutoff membranes, mitigation of end-product and non-productive inhibition of enzyme action is achieved [24, 25].

## Materials and methods

### Enzyme

All digestions were carried out using Cellic^®^ CTec3-HS from Novozymes (now Novonensis: https://www.novonesis.com/en/biosolutions/bioenergy/ethanol/cellic-ctec3-hs). As received, the enzyme preparation had a total protein concentration of 364 mg/mL as determined by Pierce BCA Assay using bovine serum albumin as a standard. As the (presumed) high concentration of stabilizers such as sugars could interfere with both the BCA assay and sugar analysis of subsequent hydrolysis experiments, the CTec3HS was desalted prior to BCA assay. The high viscosity of the CTec3HS precluded direct desalting by Size Exclusion Chromatography, as the material essentially moved as a plug through the column. To mitigate this result, a tenfold volumetric dilution (5.0 mL enzyme + 45 mL buffer) of CTec3HS in 50 mM citrate buffer containing 0.02% NaN_3_ was desalted over a HiPrep 10/26 desalting column (GE Life Sciences) in five runs of 9 mL each (45 mL total) using the same buffer as mobile phase. Samples were fractionated using an AKTA Explorer (GE Life Sciences) with automated injection, elution, and fraction collection. Fractions were collected in 5 mL aliquots into 50 mL conical tubes, with all nine runs of each desalting fractionated into the same set of collection tubes. The high molecular weight fractions from five desalting runs were pooled, yielding a total HMW (High Molecular Weight) pooled fraction volume of 100 mL, adding an additional 2.2-fold dilution (100 mL final/45 mL starting volume). The low molecular weight fractions were pooled for use in cofactor add-back studies, though no analytical efforts to determine the LMW (Low Molecular Weight) components were performed. The LMW pooled fractions totaled 200 mL for each set of five desalting runs so for any add-back studies, twofold more LMW pooled fraction than desalted enzyme was added.

The desalted CTec3HS was used in preliminary centrifugal concentrator experiments. To mitigate potential loss of activity in the desalted enzyme due to removal of the stabilizers, freshly desalted material was prepared for each experiment. To determine protein concentration, the desalted CTec3HS was diluted an additional 13.5-fold in 50 mM citrate buffer (pH 4.5) prior to the BCA assay for a total dilution of 300:1. The CTec3HS prep had roughly 300 mg/mL protein concentration based on previous lab work with other batches and the 300-fold total dilution was estimated to yield a final concentration of approximately 1 mg/mL.

The as-received CTec3HS enzyme formulation was used directly in the stirred cell experiments; however, its high viscosity made accurate low volume dispensing difficult. As a result, all enzyme additions to stirred cell experiments from stock (not diluted or desalted) were by mass based on a density of 1.42 g/mL, determined gravimetrically using 10 mL class A volumetric flasks.

### Substrate

All digestions were carried using DMR pretreated corn stover biomass prepared as described previously [26]. Briefly, a 10% (w/v) slurry of ¼” knife-milled corn stover was treated with NaOH (70 kg/ton) at 90 °C for 2 h, drained and washed with H_2_O, and then milled in a commercial disk refiner (Andritz) followed by defibrillation in a Szego mill. The DMR corn stover was washed extensively with water and glucan and xylan content were determined using NREL’s standard Laboratory Analytical Procedure [27]. After correcting for moisture, the biomass was loaded on a dry mass basis and the enzyme loading determined based on the measured glucan content. Theoretical sugar yields were determined as a function of the calculated cellulose and xylan content assuming all cellobiose/glucose and xylose in the compositional analysis came from cellulose and xylan, respectively. Conversion factors of 1.11 g glucose per g glucan, 1.05 g glucose per g cellobiose and 1.14 g xylose per g xylan were used to correct for the water gain during hydrolysis.

### Bench-scale enzyme hydrolysis

Two different bench-scale diafiltration enzyme hydrolysis systems were utilized in this work. Discontinuous sugar removal experiments were carried out at the 20 mL scale in conical centrifuge tubes. Continuous sugar removal digestions were carried out in diafiltration stirred cells at 200 mL working volume. Biomass loadings were calculated on a dry mass basis. All enzyme loadings were calculated on a milligram of protein per gram of glucan basis.

#### Discontinuous digestions

Several variations of 50 mL conical tubes were tested for leakage during incubation and Corning tubes with Centricon caps were the most consistently low loss (data not shown). The tare weights of the empty tubes (without caps) were determined and then the tubes were loaded with biomass, 1.0 mL 1.0 M citrate buffer + 0.02% NaN_3_ pH 4.5, pooled LMW fractions from the CTec3HS desalting (for cofactor add-back studies), and desalted/diluted CTec3HS. The digestion mix was brought to a final net mass of 20.0 g with dH_2_O. Initial 200 mL t0 samples were taken immediately after setting up. The samples were centrifuged in microfuge tubes at 20,800 × g for 15 min and the clarified supernatant was transferred to low volume HPLC vials. After sampling, 200 mL of 1X or 1X + (containing 22.5 mL/L LMW fraction) citrate buffer was added to the tubes to replace the sampled volume. The tubes were incubated in a rotating (inverting) mixer at 50 °C at ~ 20 rpm. Tubes were place at ~ 45° angle to allow for clearance during rotation. For timepoint sampling, tubes were removed from the rotator and centrifuged for 15 m at 4200 × g in a swinging bucket rotor. Enzyme-only and enzyme + LMW fraction-only control tubes were not centrifuged. Two hundred mL samples were taken and diluted into 800 mL dH_2_O prior to HPLC analysis for sugars. After replacing the sampled volume with 1X or 1X + citrate buffer, the tubes were mixed and returned to the incubator (enzyme-only, enzyme + LMW fraction-only, and biomass-only controls). For diafiltration digestions, the supernatants were decanted into 20 mL 10 kDa PES membrane centrifugal concentrators (Sartorious) and centrifuged at 4300 × g until the retentate was ~ 1.0 mL. The concentrated digestion mix was removed from the concentrator and returned to the digestion tube. The concentrator was rinsed with aliquots of 1X or 1X + citrate buffer, returning the rinse buffer to the digestion tube until the net digestion mass was returned to 20.0 g. The permeate was discarded and the assembled and capped concentrators were stored at 4 °C until the next sampling point.

Sugar analyses were carried out on a BioRad HPX-87H column thermostatted at 55 °C with 0.6 mL/min 0.01% H_2_SO_4_ as mobile phase and RI detection of peaks. All digestion samples were run in duplicate, including enzyme- and biomass-only controls. Theoretical conversion yields were based on sugar released compared to starting levels of glucan (Eq. 1) and xylan (Eq. 2) determined by compositional analysis. The fractional glucan and xylan content of the DMR stover was used to calculate mass of glucan and xylan in the loaded biomass. The glucan and xylan content were converted to theoretical sugar monomer content by accounting for the addition of water assuming 100% hydrolysis, i.e., 1.000 g of glucan produces 1.111 g of glucose and 1.000 g of xylan produces 1.136 g xylose. The conversion factors of 1.111 and 1.136 account for the addition of water during hydrolysis determined by the molecular weight (MW) of the free monomer divided by the MW of the monomer in the polymer; 180/162 = 1.111 (glucan→glucose), 150/132 = 1.136 (xylan→xylose). Cellobiose, if present, was converted to glucose using 1.053 as the conversion factor ((2 × 180)/362 = 1.053) and included in the glucan conversion:

1 $$\% glucose yield=\left(\frac{glucose+\left(cellobiose*1.053\right)}{glucan}\right)*100$$

2 $$\% xylose yield=\left(\frac{xylose}{xylan}\right)*100$$

For Eqs. 1 and 2, the sugar values are in mg/mL as measured by HPLC. The glucan and xylan values are in mg/mL as calculated from fractional compositional analysis and total reaction volume.

#### Continuous digestions

Initial attempts at continuous hydrolysis and diafiltration using 200 mL volume Millipore stirred cells suffered continual issues with leakage as the sealing gaskets failed under the low flow rate (4 mL/h) and resulting low pressure used to introduce diafiltration buffer. This system appears to rely on internal pressure to seal the gaskets, as there was no way to tighten the lids past a preset limit. Switching to 200 mL Sterlitech flow cells that used O-rings and screw-down bases and lids allowed manually adjustment of the sealing force to ensure a good seal. The stirred cells were equipped with 10 kDa MWCO PES Biomax membranes. Initial buffer feeding efforts using an external buffer reservoir pressurized with air or N_2_ metered through a mass flow controller were wildly inconsistent, A syringe pump was used to correct this issue. The original stirred cell design used a magnetic stir bar suspended by a spindle connected to the stirred cell cap through a plastic bushing/bearing. It became apparent after several days of continual operation that dirt of fine particulates from the biomass was abrasively wearing the bushing, resulting in “wobbling” and inconsistent operation of the stir bar. To address this, the suspended stir bar rod was removed and a stir bar was placed directly on the bottom of the stirred cell above the membrane. A 1 mm thick 70 mm pore size polypropylene felt filter disk was cut to fit just inside the stirred cell chamber and placed between the membrane and stir bar to prevent damage to the membrane.

DMR biomass was loaded into the stirred cells at 10% (w/v) solids. Ctec3HS was loaded on a mg protein/g glucan basis, either 5.0 or 2.5 mg/g depending on the experiment. The digestions were brought to 200.0 g total net mass with 50 mM citrate buffer pH 4.8 + 0.02% NaN_3_ to prevent microbial growth. Each stirred cell was equipped with a barbed inlet port and a 75-psi pressure relief valve in the lid. The permeate outlet port in the bottom was connected to the inlet port using a short section of tubing to effectively seal the system. Batch hydrolysis controls were loaded identically into 250 mL Schott bottles. After setting up the hydrolysis reactions and taking t0 samples, the stirred cells and batch bottles were incubated for 48 h in a shaking incubator at 50 °C. This setup allowed the digestion mix to liquify enough for the stir bars to be usable.

**Fig. 1:**
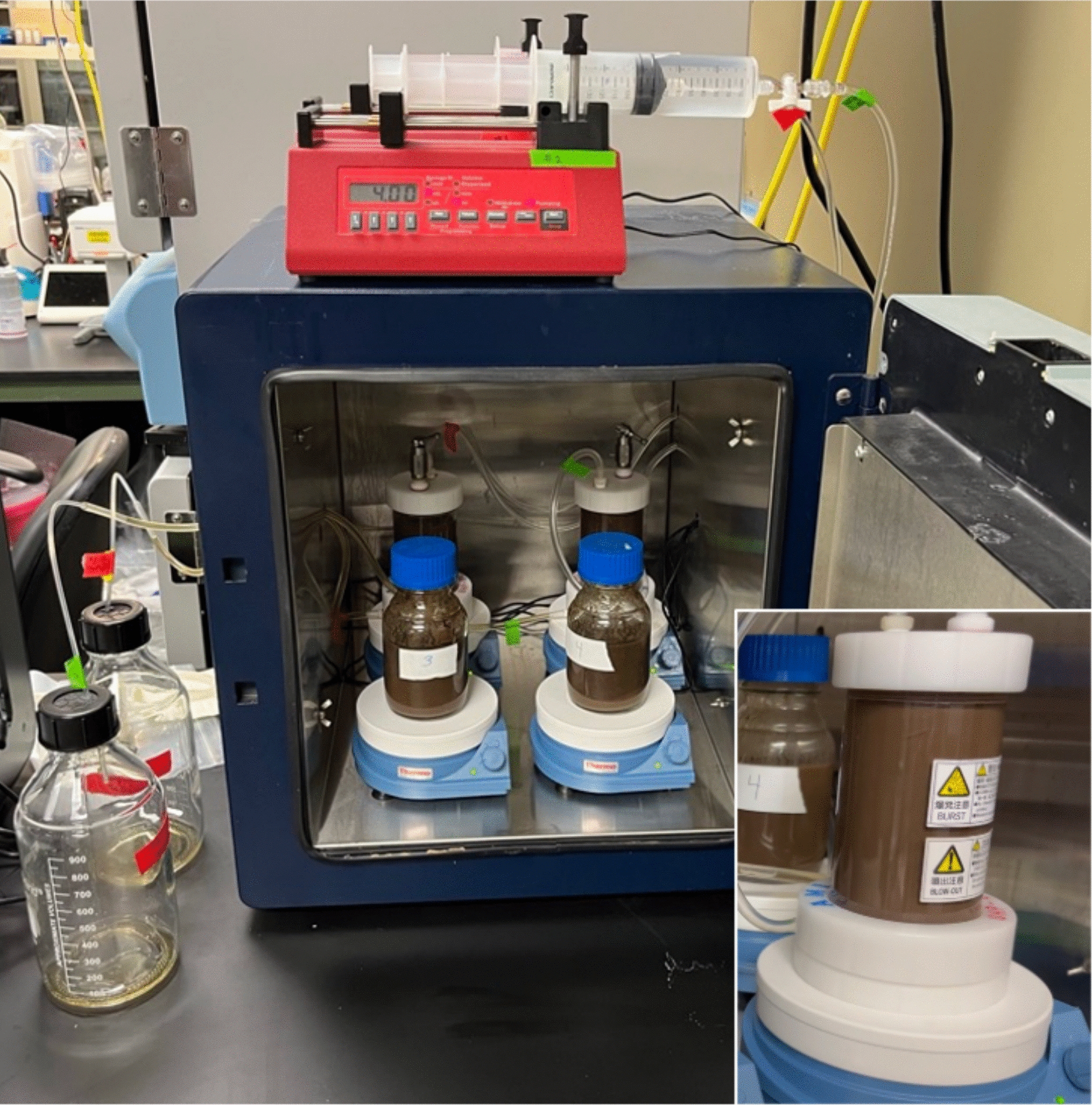
Basic diafiltration set up for continuous enzyme hydrolysis showing the incubator, syringe pumps, stirred cells (rear of incubator), batch bottles (front of incubator), stir plates, and permeate collection bottles. The 3D printed locating collar for the stirred cells is shown in the insert. Note the incubator was modified with holes in the side walls to allow for buffer inlet tubing from syringe pumps into the top of the stirred cells and permeate outlet tubing from bottom of cells to collection bottles

When the digestion was “stirrable”, the reactors were transferred to a 50 °C forced air incubator equipped with stir plates. For the stirred cells, inlet and outlet tubing was routed external to the syringe pumps and permeate collection bottles, respectively, through ports in the incubator sides (Fig. 1). The stirred cells inlet ports were connected to syringe pumps equipped with 140 mL plastic syringes (120 mL buffer volume) using Tygon tubing. The syringes were fitted with stop cocks to allow for easy removal and refilling without introducing air into the system. 50 mM citrate buffer pH 4.8 containing 0.02% NaN_3_ was pumped into the stirred cells at 4 mL/h. The syringes were refilled daily with the starting and ending volumes noted. Each permeate collection bottle was pre-tared to determine net mass of permeate for each timepoint. Citrate buffer was flowed through each cell using a syringe pump at a flow rate of 4 mL/h (~ 100 mL per d). Samples were taken from the permeate bottles at the noted timepoints and analyzed by HPLC for sugar content. Permeate bottles were mixed before sampling as significant stratification was noted early in the digestions during initial high sugar release rates. Samples were also taken from the stirred cells directly during the 5 mg protein/g glucan hydrolysis experiment. For the extended 2.5 mg protein/g glucan hydrolysis experiment, only the final timepoint included sampling directly from the stirred cells. The time course of hydrolysis was only measured using permeate samples for this latter experiment.

## Results

Hydrolysis data are available from the author’s contingent upon data dissemination policies of NREL and DOE.

### Bench-scale digestions

**Fig. 2:**
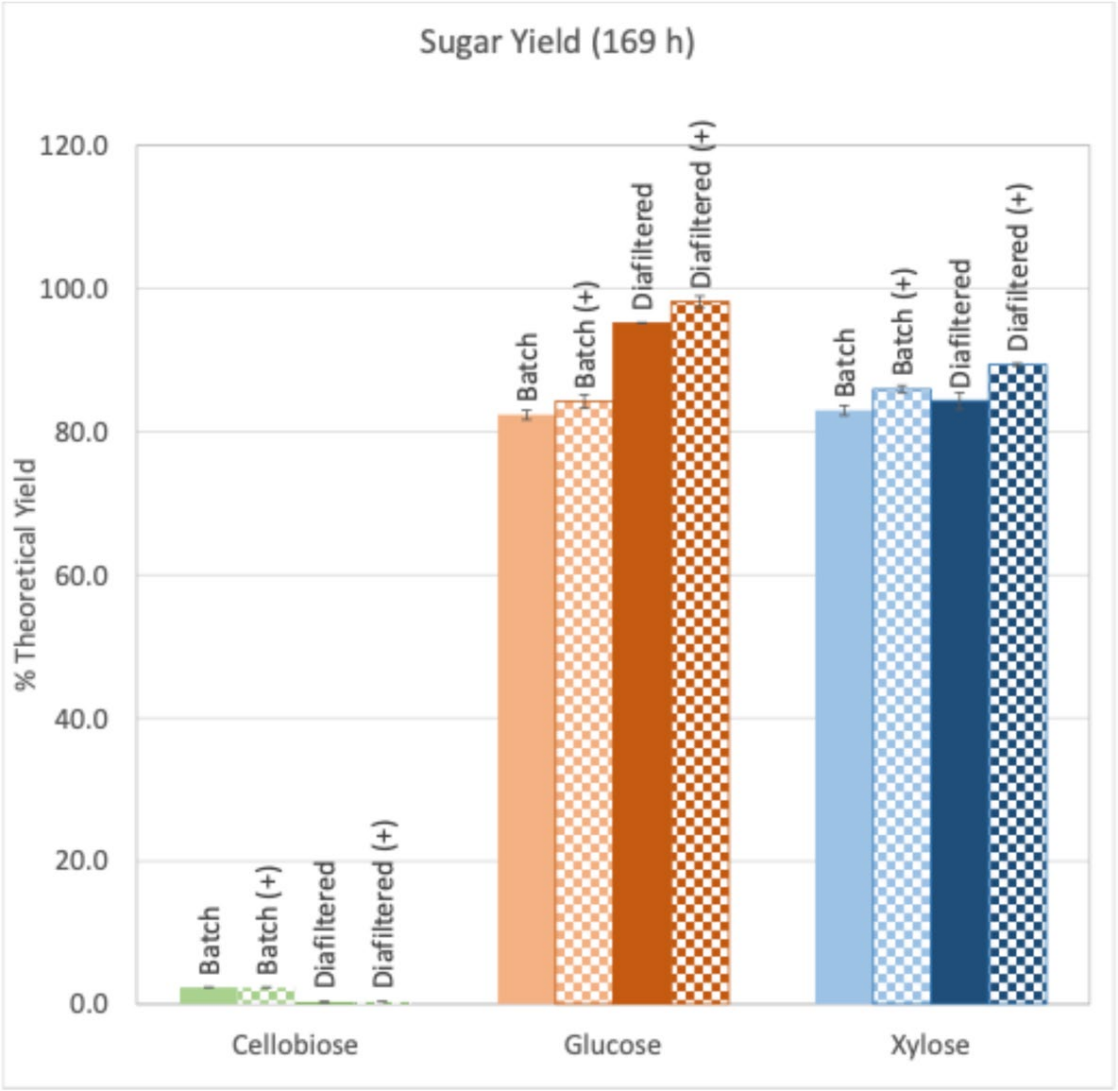
Cumulative sugar release from DMR (Deactelyated Mechanically Refined) stover using CTec3HS under sugar removal (Diafiltered) or no sugar removal (Batch) hydrolysis conditions using tube reactors and periodic diafiltration. The ((+)/checked bars) indicate the addition of the low molecular weight fraction of CTec3HS presumably containing the co-factors needed by oxidative enzymes in the commercial formulation

Novozymes Cellic^®^ CTec3HS was evaluated for sugar end-product inhibition relief using small-scale ultrafiltration in centrifugal concentrators to periodically remove sugars from the hydrolysis while retaining enzyme and biomass in the digestion mix. Periodically, the digestion tubes were centrifuged to separate the solids and liquids, and the supernatants were ultrafiltered, passing sugars into the permeates. The retentate, containing the enzymes and higher molecular weight compounds, was transferred back to the digestion tubes and the removed permeate volume was replaced with buffer. Batch control digestions were run in the same tubes and conditions but without sugar removal. Both diafiltration and batch hydrolyses were carried either with ( +) or without (−) the low molecular weight fraction of CTec3HS to evaluate the impact of oxidative enzyme co-factors on the efficacy of CTec3HS under each condition, as noted in Fig. 2.

Results from the 20 mL hydrolyses indicate that the removal of sugars during the digestion resulted in a significant increase in cellulose conversion yield. Diafiltration with desalted CTec3HS increased the yield from 82.3% to 95.2%. When the LMW fraction was added in, diafiltration increased cellulose conversion yields from 84.2% to 98.1% of theoretical. Diafiltration with and without the LMW CTec3HS components yielded ~ 15.6% and 16.5% increase in glucose release, respectively, compared to batch hydrolysis alone (Fig. 2). Increases in xylan conversion were comparatively minor, increasing from 82.9% to 84.3% and 85.9% to 89.4% for hydrolyses without and with the LMW fraction, respectively. These represent increases of 1.6% and 4.0%, respectively. In addition, the batch hydrolyses both contained cellobiose at just over 2% theoretical cellulose yield levels, while the diafiltered hydrolyses cellobiose levels were < 0.5%.

**Fig. 3:**
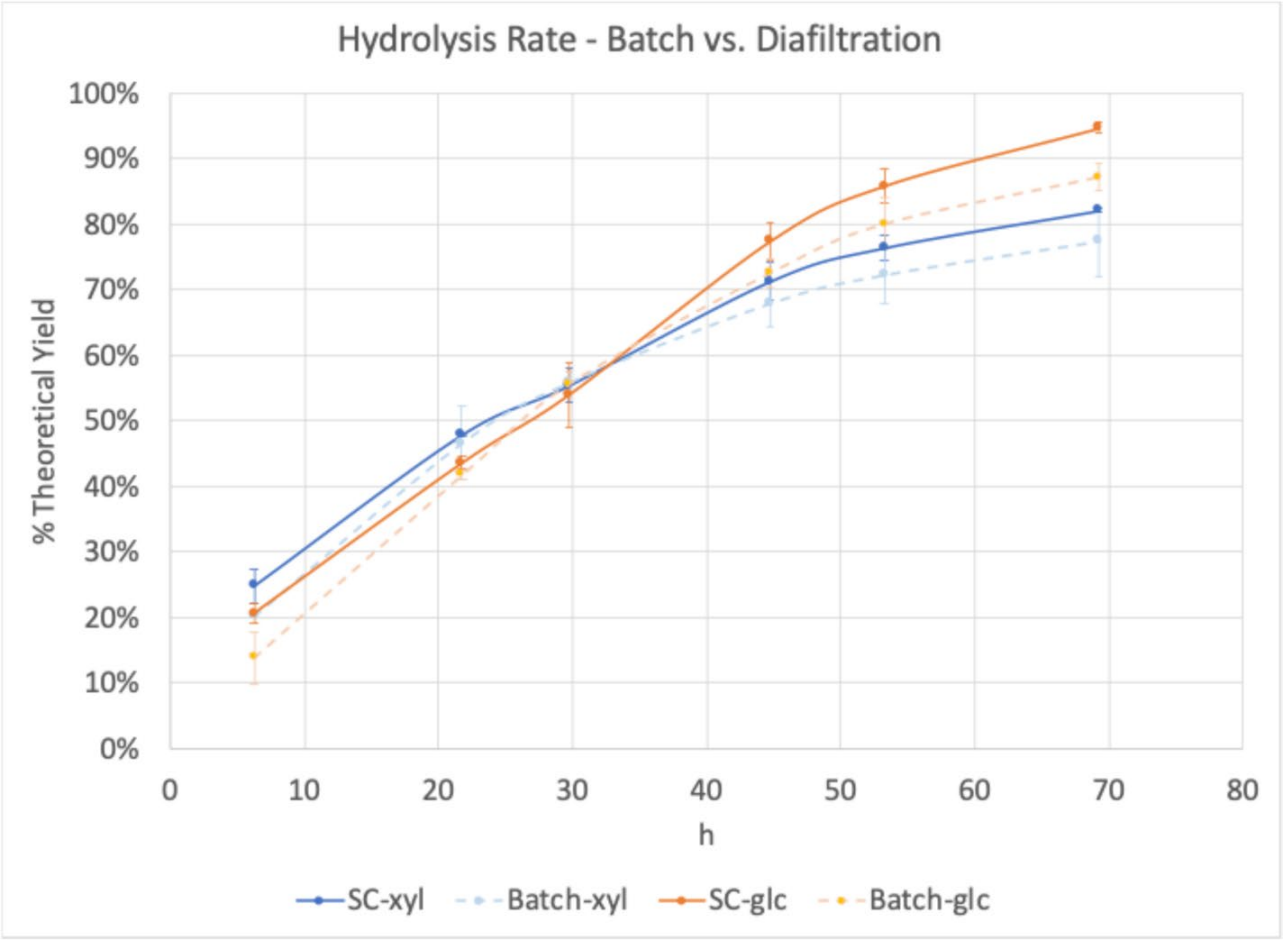
Hydrolysis by diafiltration in stirred cell (SC) at 5 mg protein/g glucan loading. Batch reactors were sampled directly, and SC reactors were sampled from both the stirred cell (retentate) and the permeate. glc = glucose, xyl = xylose

**Fig. 4:**
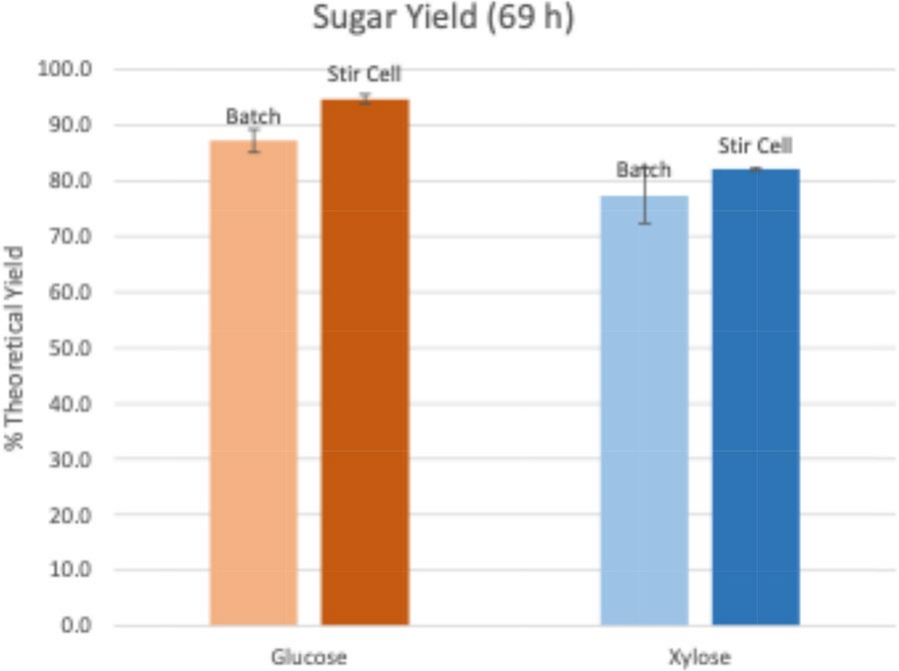
Final yields of 200 mL bench-scale stirred cell digestions comparing batch and continuously filtered enzyme digestions. (5 mg/g Ctec3HS)

To test a more continuous enzyme hydrolysis operation, the digestions were scaled up from 20 mL tube reactors to 200 mL stirred cells. Sampling directly from the stirred cells was mechanically problematic and resulted in somewhat irregular digestion curves (Fig. 3) so follow on stirred cell digestions were sampled only from the permeate during the hydrolysis. After 69 h of hydrolysis, the stirred cell hydrolyses reached 95% and 82% glucose and xylose theoretical yield, respectively. This was significantly higher than the batch yields of 77% glucose and 82% xylose (Fig. 4).

**Fig. 5:**
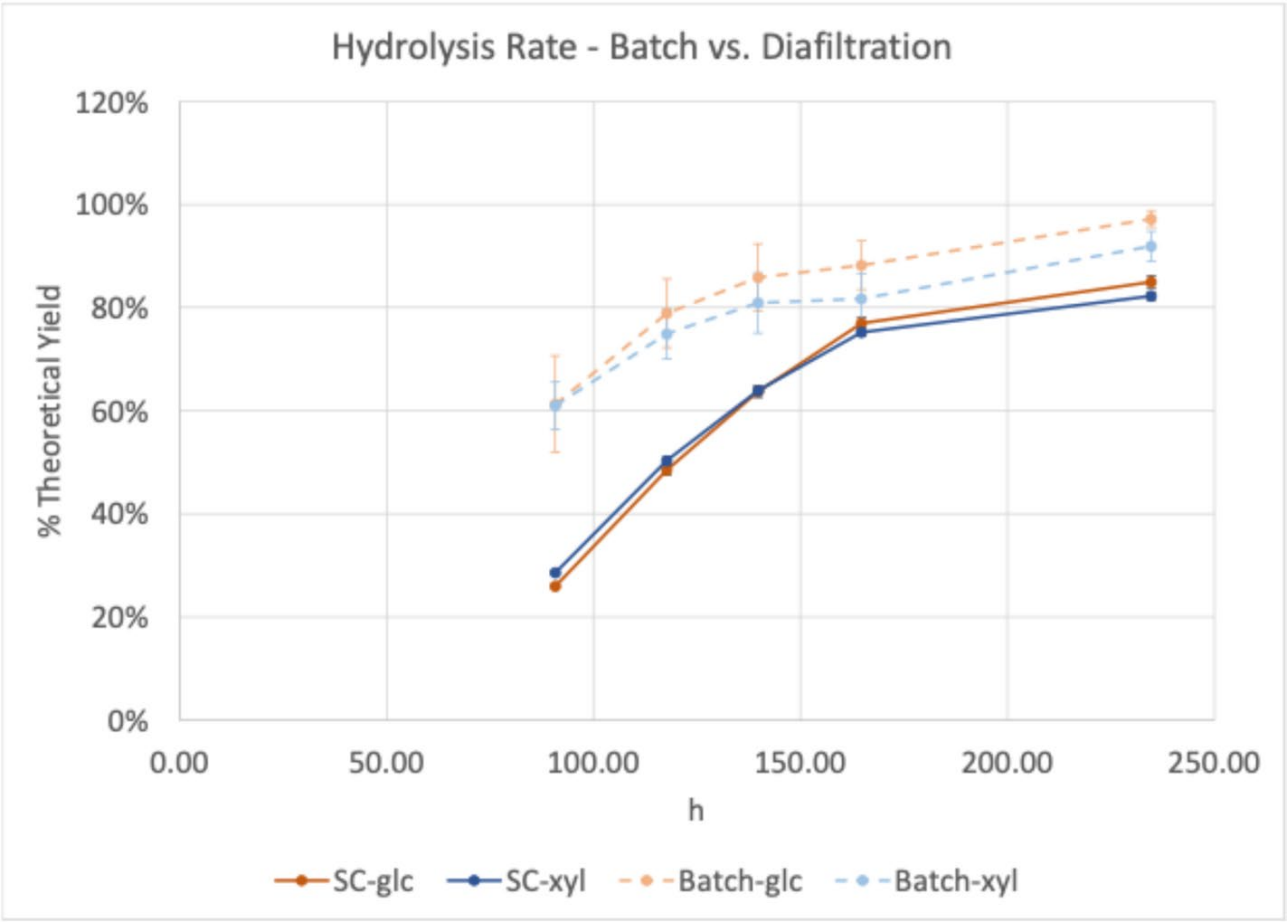
Extended hydrolysis by diafiltration in stirred cell (SC) at 2.5 mg protein/g glucan loading. Batch reactors were sampled directly, and SC reactors were sampled only from the permeate, so yields indicated are incomplete as sugars in the retentate are not quantified. glc = glucose, xyl = xylose

Considering the relatively high yields over a short hydrolysis (< 3 days), the same system was tested at 2.5 mg enzyme/g glucan, 50% of the original enzyme loading. For this experiment, the digestion was allowed to proceed for an extended time. Stirred cells were not sampled directly, only from the stirred cell permeate collection bottles and the batch reactors. While this sampling regimen allowed monitoring of the batch hydrolysis directly, the stirred cell digestion could only be monitored indirectly, as a significant fraction of the released sugars at any given time remained in the stirred cell. This apparent lag in conversion is illustrated in Fig. 5.

**Fig. 6:**
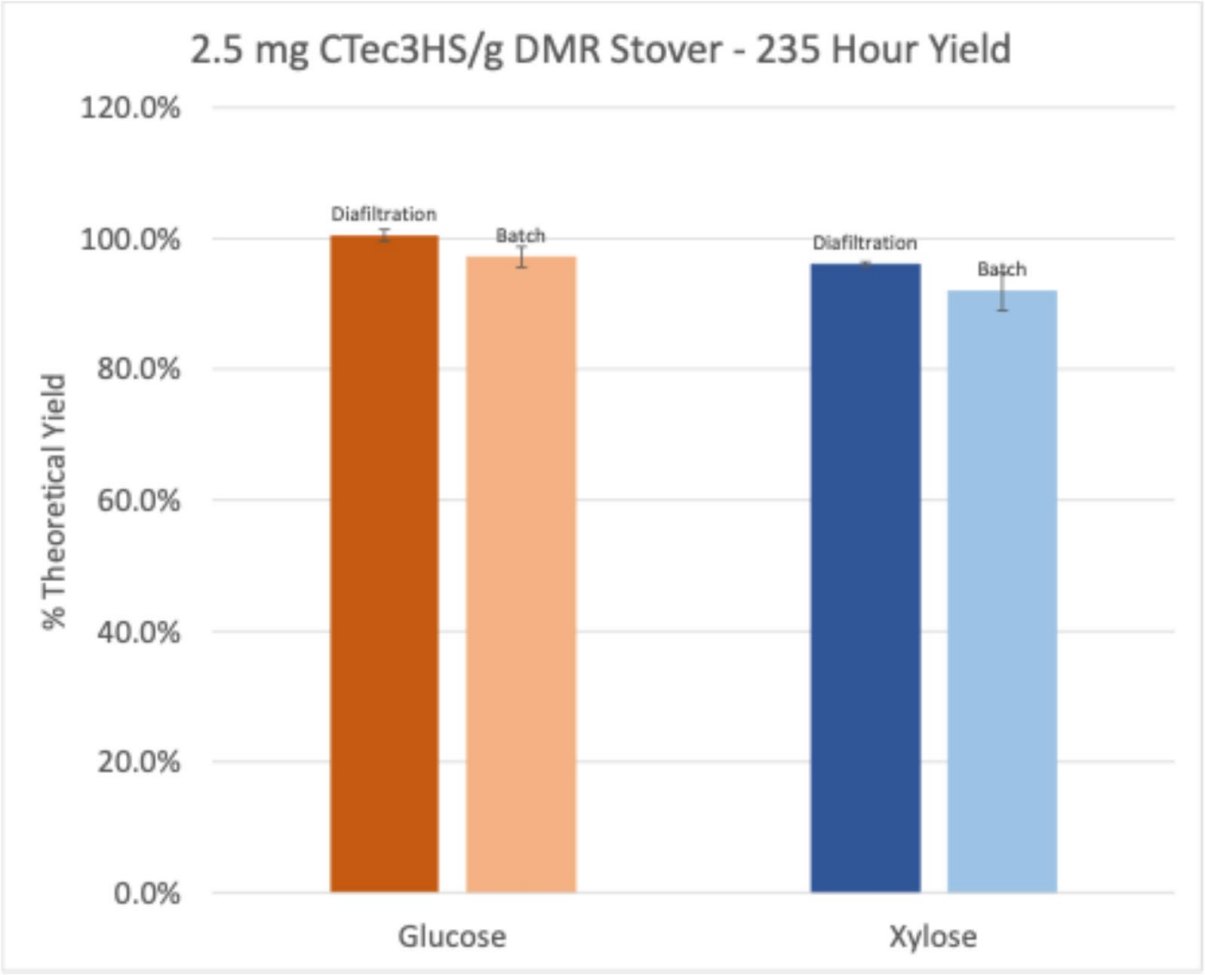
Final sugar yields from hydrolysis of Deacetylated Mechanically Refined (DMR) corn stover at an enzyme loading of 2.5 mg protein/g glucan. For diafiltration hydrolysis, yields were calculated, including sugars from final retentate and total permeate

After 235 h, the experiment was terminated and the retentate of each stirred cell was analyzed for sugar concentrations, allowing us to calculate the total final conversion yield. Glucose yields for stirred cell and batch hydrolyses were 100% and 97%, respectively. Xylose yields were slightly lower at 96% (stirred cell) and 92% (batch) (Fig. 6).

## Discussion

Historically, EH is carried out batchwise; however, there is increasing interest in applying process intensification to improve this rate limiting unit operation by enabling it to be carried out in a continuous manner. The primary advantage of continuous enzyme hydrolysis being the removal of sugar end products which are known to cause powerful inhibition of cellulases and xylanases [24, 28–30]. An additional obvious application of CEH technology is the production of sugars in separate hydrolysis, overcoming the well-documented mismatch in pH and temperature for commercial cellulase formulations and yeast or *Zymomonas* fermentations [12]. Fermentation is typically shorter compared to EH as well, necessitating larger EH tankage to match a fast sugar feed rate. While the classic Simultaneous Saccharification and Fermentation (SSF) process solved the issue of end-product inhibition, the mismatch in pH and temperature optima between EH and fermentation even further reduced the hydrolysis rate [31]. Clearly, an ability to conduct saccharification and fermentation at their respective optimal conditions is critical. However, doing so removes the ability to benefit from sugar product removal during SSF, i.e., the “SSF effect” and reduces enzyme efficacy, requiring higher loadings and/or longer hydrolysis time [31]. Therefore, CEH is conceptually viable in this case as both temperature and pH can be controlled to the optimum for the enzymes, while inhibitory sugars are removed to foster increased rates and conversion extent.

Most biomass sugars fermenting yeast, such as CFUELS from Lallemand, Inc. and Clariant’s C5/C6 fermenting yeast function near optimally at the pH recommended for commercial enzyme formulations, such as Novozymes Cellic^®^ CTec3 HS. However, and regarding the point made above, the temperature optima for the enzyme formulations and commercial ethanologens is more difficult to match. Industrial fungal hydrolytic formulations function optimally at 45–60 °C [12] and reports of thermophilic ethanologens have been very limited and not commercialized. Although a better fit than *Zymomonas*, yeast fermentations could also benefit from full or partial saccharification of pretreated feedstock solids by allowing saccharification to proceed at 50 °C in CEH. In the case of yeast ethanologens, the SSF effect is more readily demonstrable.

A primary application for this work is the use of EH used to produce sugars from lignocellulose as the final product rather than directly converting them via fermentation. While SSF provides a means of removing inhibitory sugars in real time, fermentation limits product options. A clean sugar stream product allows catalytic conversion or use in highly controlled fermentation requiring clean sugar feedstocks; however, economics dictates the enhanced rates, extents of conversion, and specific efficacy (minimal enzyme usage) of CEH. An example of this new application is the Sugar Depot concept (Reyhaneh Shenassa, BETO, 2022). In the Sugar Depot context, the process would consist of pretreatment, CEH, and sugar stabilization for storage, with fractionated lignin stream(s) available as co-products for additional valorization.

Advantages of continuous enzyme hydrolysis-based sugar production include: enabling a continuous lignocellulose biomass manufacturing platform; reducing sugar product feedback inhibition on the EH reaction to achieve higher sugar production rate, titer, and recovery yields, higher productivity; reduce enzyme loading by retention/recycling of hydrolytic enzymes bound to the solids to reduce costs, facilitating downstream upgrading strategies that require clarified, desalted sugar streams (i.e., aqueous inorganic catalysis and lignin streams unadulterated by polyelectrolyte flocculants); and consolidation of unit operations required to produce a concentrated clarified sugars stream and reduce CAPEX. Continuous Enzymatic Hydrolysis aims to reduce greenhouse gas (GHG) emissions of SAF and bio-based chemicals production by increasing sugar–lignin platform processing efficiency, improving the economics, decreasing the carbon footprint of enzyme production and transport through reduced enzyme use, and minimizing scale-up risks of biorefinery sugar–lignin production through development of a deployable CEH process.

## Conclusions

Bench-scale experiments indicate that the CEH process can enhance enzymatic hydrolysis efficiency by 10–15% at a given timepoint compared to batch processes. A significant breakthrough of the CEH process is its ability to achieve equivalent endpoint conversions with approximately 50% less enzyme loading. This reduction in enzyme requirement not only lowers operational costs but also contributes to the sustainability of the process. Moreover, the specific configuration of reactor-membrane units in CEH boosts conversion, further enhancing the process's economic viability. Finally, the ability to produce soluble sugar streams from saccharification removes the long-standing requirement to conduct SSF, which greatly ensures semi continuous, if not continuous processing of feedstock at commercial scale.

Overall, this work aimed to revolutionize the production of soluble clarified biomass sugars and insoluble lignin-rich streams in Gen2 biorefineries. Overcoming the limitations of existing process technologies such as SSF via this innovative approach could significantly reduce costs and increase the efficiency of these biorefineries. This advancement is a critical step towards the broader adoption of renewable energy sources and the sustainable production of biomaterials, marking a significant stride in the field of enzymatic hydrolysis technology.

## Data Availability

No data sets were generated or analyzed during the current study.
